# Is Kidney-Ureter-Bladder Radiography Still a Helpful Tool to Address Acute Ureteral Colic in Emergency Settings?

**DOI:** 10.7759/cureus.90365

**Published:** 2025-08-18

**Authors:** Mohammad Alkarajeh, Faris Abushamma, Mahfouz Ktaifan, Abdoh Abdallah, Amir Aghbar, Maha Akkawi, Mosab Maree, Sa’ed H. Zyoud

**Affiliations:** 1 Department of Nephrology, An-Najah National University Hospital, Nablus, PSE; 2 Department of Medicine, Faculty of Medicine and Allied Medical Sciences, An-Najah National University, Nablus, PSE; 3 Department of Urology, An-Najah National University Hospital, Nablus, PSE; 4 Department of Pediatrics, An-Najah National University Hospital, Nablus, PSE; 5 Department of Medical Imaging, University of Western Ontario, London, CAN; 6 Department of Clinical and Community Pharmacy, Faculty of Medicine and Allied Medical Sciences, An-Najah National University, Nablus, PSE; 7 Clinical Research Center, An-Najah National University Hospital, Nablus, PSE

**Keywords:** acute ureteral colic, kub radiography, noncontrast ct scan, ureteric stone, ureteroscopy

## Abstract

Background

This study aims to identify the reliability of kidney-ureter-bladder (KUB) radiography as a triage tool in acute ureteral colic (AUC). Moreover, this article correlates between KUB and non-contrast computerized tomography (NCCT) in view of stone characteristics and clinical outcomes.

Methodology

A retrospective cohort study recruited patients who had proven ureteric stones on NCCT. A blinded review of KUB and NCCT was performed to identify the following variables in both tests: site, ureteric stone maximum diameter, and stone density. Correlation between KUB radiography and NCCT has been performed. The intermethod reliability was used to measure the degree to which test scores are consistent when the methods or instruments employed vary.

Results

One hundred fifty-one patients were included, of whom 75 (50%) had negative KUB and positive NCCT results for ureteric stones based on the blinded review. Lower ureteral calculi were found to be the most common location in both KUB (*n *= 49, 65%) and NCCT images (*n *= 81, 54%). The median stone diameters of KUB and NCCT were 5 (3-8) mm and 6 (4-9) mm, respectively. Hounsfield unit densities of more than 630 were found in 86 (57%) patients, and radiopaque stones were found in 76 (50%) patients. There was moderate and significant concordance (Cohen’s kappa = 0.520) between NCCT and KUB regarding stone location (*P *< 0.01). There was a strong concordance (Cohen’s kappa = 0.804) between NCCT and KUB in detecting ureteric stone maximum diameter (*P *< 0.01). Stone density was weakly correlated between KUB and NCCT (Cohen’s kappa = 0.254) (*P *= 0.001). Thirty-four cases (45%) of negative KUB results required surgical intervention (SI). Sepsis (*n *= 5, 15%) and acute kidney injury (*n *= 23, 68%) were the main indications for SI in negative KUB and positive NCCT ureteric stones.

Conclusions

KUB radiography should not be used as a triage tool in AUC due to potentially harmful outcomes. However, KUB radiography can be reliably used during follow-up, as there is a strong correlation between KUB radiography and NCCT for KUB-detectable ureteric stones.

## Introduction

Noncontrast computerized tomography (NCCT) has become the standard method for diagnosing ureteric stones in emergency settings [1]. Traditionally, kidney-ureter-bladder (KUB) radiography, intravenous urography (IVU), and ultrasound (US) have been used to diagnose acute ureteral colic (AUC) with variable sensitivity and specificity. For example, US is a highly sensitive test for kidney stones and hydronephrosis but has low sensitivity for identifying ureteric stones [2]. Furthermore, IVU was previously considered the gold standard method for diagnosing ureteric stones, but NCCT has replaced it globally [3,4]. NCCT offers an accurate assessment of stone size, site, and density in addition to a higher detection rate of secondary signs such as perinephric stranding, ureteral dilatation, and stone impaction markers [4,5]. KUB radiography is still considered a triage tool in the emergency department for patients with possible AUCs in developing countries. The justification for using KUB radiography is its low cost, low radiation exposure, and availability in all centers.

Nevertheless, the sensitivity and specificity of KUB are 44%-77% [6]. Levine et al. reported that the accuracy of the initial KUB reading in an emergency setting was 40% [7]. Thus, using KUB radiography as a sole tool for investigating the AUC is unreliable and may lead to adverse outcomes if ureteric stones cause complications such as infection and acute kidney injury (AKI). Merging US and KUB radiography increases the sensitivity and specificity of detecting ureteric stones in emergency settings [4]. However, US is not always quickly accessible, and it is operator-dependent [8,9]. Thus, such a combination is not always possible or optimal in an emergency setting.

Despite the widespread use of KUB radiography in the emergency setting to diagnose AUC, there is a lack of evidence supporting its effectiveness and relevance in clinical practice. Therefore, we investigated the accuracy of KUB radiography for detecting NCCT-proven ureteric stones. This study aimed to assess the reliability of KUB radiography as a triage test in terms of the AUC. Furthermore, the accuracy of KUB-based stone characteristics was assessed in comparison to that of NCCT. The results of this study will directly impact AUC management in developing countries where NCCT is not always accessible or feasible.

## Materials and methods

Study design and setting

A retrospective cohort study obtained clinical and radiological data for patients diagnosed with AUC at our tertiary urology center between April 2020 and May 2021. Patients with AUC always come to the emergency department, where KUB radiography and blood tests are performed. Afterward, based on the emergency physician's decision, NCCT is ordered for some cases with high suspicion of ureteric stones.

Inclusion and exclusion criteria

NCCT-proven ureteric stone patients who presented with AUC were included. Children and complicated stones at first encounter (fever and AKI) were excluded.

Study population, sampling, and data collection

We reviewed all patients who were diagnosed with ureteric stones based on NCCT. NCCT was performed using a Canon AQ Prime SP device, with the scan starting from the dome of the diaphragm and ending at the level of the lesser trochanter of the femur. At the same time, the patient is supine and proceeds in a helical fashion and craniocaudal direction. Reconstruction was performed by the device, in which the thickness and spacing were 1 mm each, and the field of view (FOV) was 40 cm. The filter convolution (FC) was 17, the kilovoltage peak (kVp) was 135, the scan FOV was 0-450 nm, the rotation time was 0.6 seconds, and the detector rows were 0.5 * 80 mm.

KUB radiography was obtained for AUC patients, as it is part of the emergency protocol in such cases. The CT scout film was also reviewed in cases of missing or poor KUB radiography.

Clinical data, KUB radiography characteristics, and NCCT features were collected. All NCCT and KUB radiographs were reviewed retrospectively in a blinded manner by a qualified radiologist to identify the following characteristics in each imaging modality independently. Inconsistency was resolved by a consensus meeting.

KUB-based radiological variables

Ureteric Stone Site

Ureteric stone site is classified into upper, middle, and lower.

Multiple Ureteric Stones

If more than one stone was found in the ureter, we considered it to be multiple ureteric stones.

Ureteric Stone Maximum Diameter (mm)

Defined as the longest diameter of the ureteric stone. The stone was measured in transverse and axial length on KUB radiography. The maximal length was obtained.

Stone Density

Density on KUB radiography was measured by comparing stone appearance to the density of the ribs. Density was classified into three categories: Level 1, where stone density was less than that of the ribs; Level 2, where stone density was equal to that of the ribs; and Level 3, where stone density was greater than that of the ribs. Radiolucent stones were classified as level 0.

NCCT-based radiological variables

Ureteric Stone Site

Ureteric stone site is classified into upper, middle, and lower.

Multiple Ureteric Stones

If more than one stone was found in the ureter, we considered it to be multiple ureteric stones.

Ureteric Stone Maximum Diameter

Measured by taking both width and length. The maximum measurement is included.

*Hounsfield Unit Density of Ureteric Stone* 

Measured by our expert radiologists to differentiate between stone consistencies based on CT measurements. Stones that had a density of 630 Hounsfield units (HUs) or less were classified into one group, and stones that had a density of more than 630 HU were classified into another group [10].

Clinical outcome

The clinical outcomes for all stones were assessed at six weeks and classified into either spontaneous stone passage (SSP) or surgical intervention (SI) because of AKI (estimated glomerular filtration rate (eGFR) <60 mL/minute/1.73 m^2^), fever, intractable pain or persistent ureteric stones.

Statistical analysis

Analysis was performed with IBM-SPSS Statistics, version 26.0 (IBM Corp., Armonk, NY). Categorical variables were presented as frequencies and percentages. In nonnormal data distributions, continuous data were provided as the median and interquartile range (interquartile range, 25th to 75th percentile). To compare baseline characteristics between the SSP and SI groups, Pearson's chi-square or Fisher's exact test for categorical variables and the Mann-Whitney U test for continuous data were used. In addition, Cohen's kappa test was used to assess the intermethod reliability for categorical variables, and the intraclass correlation coefficient was used to assess the intermethod reliability for continuous variables. The intermethod reliability was used to measure the degree to which test scores were consistent when the methods or instruments employed varied (Cohen's kappa: 0.60-0.79 moderate; 0.80-0.90 strong). A *P*-value of <0.05 was considered statistically significant [11].

## Results

Patient demographics and clinical presentation

We examined 287 AUC records and found that 151 patients met the criteria to be included in our study. Among these patients, 115 (76%) were male, and the median age of the group was 44 years, with a range of 32 to 60 years. Diabetes was present in 24 patients (16%). Table 1 provides additional information about the patients' characteristics and medical conditions.

**Table 1:** Patient demographics and clinical presentation.

Demographics	Frequency (%) or median (Q1-Q3) (*N *= 151)
Patient gender	
Female	36 (23.8%)
Male	115 (76%)
Patient age (years)	44 (32-60)
Site of pain	
Right	73 (48.3)
Left	81 (51.7)
Diabetes mellitus	24 (15.9)

KUB radiography detection rate of ureteric stones

The detection rate of stones based on KUB radiography was 75 (49.7%). Levels 2 and 3 stone densities were seen in 69 patients (46%). Table 2 shows the distribution of KUB densities according to the level.

**Table 2:** KUB density levels. KUB, kidney-ureter-bladder

Density levels	Frequency (%)
0 (radiolucent)	75 (49.7)
1 (density less than the density of the ribs)	7 (4.6)
2 (density equal to the density of the ribs)	40 (26.5)
3 (density more than the density of the ribs)	29 (19.2)

KUB- and NCCT-based radiological variables

Table 3 shows the radiological findings of KUB radiography and NCCT.

**Table 3:** Correlation between KUB radiography and noncontrast CT. *N = 151. ^a^Interrater reliability and statistical significance were calculated using Cohen's kappa test. ^b^Interrater reliability and statistical significance values were calculated using the intraclass correlation coefficient. KUB, kidney-ureter-bladder; HU, Hounsfield unit

Radiological variables	KUB, frequency (%) or median (Q1-Q3) (*N *= 76)	CT-scan, frequency (%) or median (Q1-Q3) (*N* = 151)	Intermethod reliability	*P*-value
Laterality			0.737^a^	<0.01^a^
Right	38 (50)	73 (48.3)		
Left	38 (50)	78 (51.7)		
Ureteric stone site			0.520^a^	<0.01^a^
Upper	17 (22.4)	55 (36.4)		
Middle	10 (13.2)	15 (9.9)		
Lower	49 (64.5)	81 (53.6)		
Ureteric stone maximum diameter (mm)	5 (3-8)	6 (4-9)	0.804^b^	<0.01^b^
					Multiple ureteric stones			0.115^a^	0.173^a^
No	72 (94.7)	126 (83.4)							
Yes	4 (5.3)	25 (16.6)							
Stone density	Level 0 + level 1: 82 (54.3)*	Density ≤ 630 HU: 65 (43)	0.254^a^	0.001^a^
	Level 2 + level 3: 69 (45.7)*	Density > 630 HU: 86 (57)		

The variables are described next.

Ureteric Stone Site

From the three categories (upper, middle, and lower), lower ureteral calculi were found to be the most common location in both KUB (*n* = 49, 65%) and NCCT images (*n* = 81, 54%). Upper and middle ureteral calculi were found in 27 patients (36%) in KUB and 70 patients (46%) in NCCT.

*Ureteric Stone Maximum Diameter (mm)*

A median ureteric stone maximum diameter of 5 (3-8) mm was measured in KUB, while a median of 6 (4-9) mm was measured in NCCT.

Multiple Ureteric Stones

When using NCCT, 25 (17%) patients were identified to have multiple ureteric stones on NCCT, whereas only 4 (5%) patients were identified to have multiple ureteric stones on KUB.

HU Density of Ureteric Stone

KUB did not identify 75 patients (50%). In NCCT, HU densities of 630 or less were found in 65 patients (43%), and HU densities of more than 630 were found in 86 patients (57%).

Correlation between KUB radiography and NCCT in view of size, site, and laterality

The correlation between KUB radiography and NCCT was examined across multiple variables (Table 3). The variables are described as follows.

Ureteric Stone Site

There was moderate concordance (Cohen’s kappa = 0.520) between NCCT and KUB in assessing the site of ureteric stones. This concordance was acceptable and statistically significant (*P *< 0.01).

Ureteric Stone Maximum Diameter

We found a strong concordance (Cohen’s kappa = 0.804) between NCCT and KUB in measuring the maximum diameter of ureteric stones. This concordance was acceptable and statistically significant (*P *< 0.01).

Multiple Ureteric Stones

The intermethod reliability between KUB and NCCT was very low (Cohen’s kappa = 0.115). However, this finding was statistically insignificant (*P *= 0.173).

Correlation between KUB radiography and NCCT in view of stone density

By comparing the two groups shown in Table 3, there was low concordance (Cohen’s kappa = 0.254) between NCCT and KUB in assessing the density of ureteral calculi. This finding was acceptable and statistically significant (*P *= 0.001).

The clinical outcome of nondetectable ureteric stones on KUB radiography

Thirty-four (45%) patients with negative KUB and positive NCCT required SI. The mean time of SI was 3.3 (1-37) days. There are several reasons for SI: sepsis (*n* = 5, 15%); worsening eGFR (<60 mL/minute/1.73 m^2^) (*n *= 23, 68%); and suboptimal response to conservative therapy (*n *= 6, 17%).

Stone location was significantly associated with SI. SI was significantly higher in the upper and middle locations (*n *= 23, 68%; *P *= 0.006). SSP was significantly higher in the lower stone location (*n *= 28; 68%; *P *= 0.006).

A median stone length of 7 (5-11.25) mm was statistically associated with SI, and a median stone length of 4 (3-6) mm was associated with SSP (*P *= 0.001). Table 4 shows a comparison of radiological variables between surgical intervention and spontaneous stone passage for KUB-negative ureteral calculi.

**Table 4:** Comparison of radiological variables between surgical intervention and spontaneous stone passage for KUB-negative ureteral calculi (includes patients who were negative on KUB radiography and positive on noncontrast CT). ^a^The bold values indicate *P*-value < 0.05. ^b^Statistical significance values calculated using Pearson’s chi-square test. ^c^Statistical significance values calculated using Fisher’s exact test. ^d^Statistical significance values calculated using the Mann-Whitney U test. KUB, kidney-ureter-bladder

Radiological variables	Group 1: Surgical intervention	Group 2: Spontaneous stone passage	*P*-value^a^
	*N*= 34 (45.3%)	*N *= 41 (54.7%)	
Laterality			0.123^b^
Right	21 (61.8)	18 (43.9)	
Left	13 (38.2)	23 (56.1)	
Ureteric stone site			0.006^c^
Upper	15 (44.1)	10 (24.4)	
Middle	8 (23.5)	3 (7.3)	
Lower	11 (32.4)	28 (68.3)	
Ureteric stone maximum diameter (mm)	7 (5-11.25)	4 (3-6)	0.001^d^
Multiple uretric stones			0.130^c^
No	28 (82.4)	39 (95.1)	
Yes	6 (17.6)	2 (4.9)	
Stone density			0.817^b^
Density ≤ 630 HU	19 (55.9)	24 (58.5)	
Density > 630 HU	15 (44.1)	17 (41.5)	

## Discussion

KUB may miss up to half of the ureteric stones in the emergency setting. Furthermore, our results showed that half of the patients who reported negative KUB results had SI because of complications. Thus, the use of KUB as a triage tool in emergency settings is questionable in the era of NCCT. Theoretically, radiolucent stones represent approximately 10% of all ureteric stones and are mainly composed of uric acid [12,13]. Thus, KUB radiography should clearly show most of the ureteric stones. However, our data show that a blinded review of KUB radiography by a qualified senior radiologist identified ureteric stones in less than half of the patients. Furthermore, preliminary KUB radiography may be unnecessary in cases of negative NCCT for ureteric stones, as Kennish et al. showed that 38% of AUC patients had unnecessary KUB [14]. Therefore, the poor sensitivity of KUB radiography for diagnosing ureteric stones and the potential for unnecessary radiation exposure justify canceling KUB radiography as a triage test for suspected AUC.

NCCT has become the gold standard method for diagnosing ureteric stones [15,16]. The advantages of NCCT over other imaging modalities are that NCCT offers an accurate assessment of stone burden and characteristics, showing stone impaction markers and signs of kidney affection, and finally, NCCT may show detailed abdominal anatomy and possible concomitant pathology [5,17,18]. Identifying those variables based on NCCT, such as extracorporeal shock wave lithotripsy (ESWL) and ureteroscopy, is also essential for choosing the best treatment modality. The main disadvantage of NCCT is the higher radiation dose in a young cohort of patients. However, low-dose NCCT can be used with a sensitivity of 93.1% (95% confidence interval (CI): 91.5-94.4) and a specificity of 96.6% (95% CI: 95.1-97.7%) [19,20].

KUB radiography is still a valid tool for use during follow-up for positive ureteric stones on NCCT. In this study, there was a strong correlation and similarity between the stone size, location, and laterality of both KUB and NCCT for detectable stones on KUB radiography. Katz et al. previously showed that ureteric stone dimensions on KUB are mostly similar to those on NCCT [20-22]. Furthermore, stone location is another important variable that can be reliably detected via KUB radiography. Therefore, in the presence of such high concordance between KUB and NCCT in view of stone size and location, KUB can be used reliably as a follow-up tool to monitor the success of stone management (ESWL), medical expulsive therapy (MET), post-ureteroscopy, and laser fragmentation. Such a concept allows physicians to use NCCT less to judge the clinical outcome of stone management but to rely on the concordance between KUB and NCCT.

It is debatable whether the HU of urolithiasis is directly linked to the type and composition of the stone. Several studies have shown that stone composition is linked to the presence of calcium-containing stones, which are greater in HU than in other compositions [23,24]. However, a different set of assemblers has shown that the sensitivity and specificity of HU for predicting stone composition are low, especially for stones less than 5 mm in length [25,26]. In our study, we utilized the HU as a means to infer the hardness and consistency of stones, given the challenges in performing physical analyses of ureteric stones. These challenges stem from the relatively small size of ureteric stones compared to those found in the kidney, the contemporary approach of removing stones with advanced laser technology such as Thulium lasers, and the difficulty in retrieving stones from patients who have passed them spontaneously. In our study, the HU density of stones on NCCT did not correlate with KUB radiography findings. It was previously established that a cutoff value of 630 HU on NCCT is ideal for predicting radiopaque stones on KUB [10]. However, our data revealed a poor correlation between the NCCT and KUB HU values. This finding is consistent with previously published evidence that NCCT underestimates the radiopacity of a stone on a plain KUB (negative predictive value (NPV) 67%) [27]. In our opinion, stone composition is the main predictor of radiopaque stones, and HU density failed to predict stone composition in vitro [28].

Using KUB radiography as a triage tool to diagnose ureteric stones in patients suspected of having an AUC may lead to missing half of the patients with a positive NCCT. Such missing data may lead to adverse outcomes in AUC patients, especially if complications occur. In our analysis, 45% of the patients in the negative initial KUB cohort underwent surgical intervention. AKI and sepsis were the main indications for surgical intervention in 31% and 7% of patients, respectively. The ureteric stone diameter and location were found to be independent factors for surgical intervention in this group. Discharging patients without a clear diagnosis may put such patients at risk of sepsis and significant AKI. Sepsis associated with an obstructed urinary tract may lead to mortality in up to 20% of patients [29]. AKI associated with an obstructed system has a direct adverse outcome and leads to urgent intervention in the form of nephrostomy or stent, which may prolong the hospital stay [30]. Therefore, because of the potential for ureteric stone complications, we did not use KUB radiography as a triage test in the emergency setting. Instead, NCCT should be the gold standard in AUC, which is supported by the American Urological Association (AUA) and the European Association of Urology (EAU) guidelines.

Strengths and limitations

The retrospective nature and absence of stone composition data are considered the main limitations. Future prospective correlations between imaging modalities and stone composition may help to clarify stone density and visibility on KUB radiography. The strength of this study was that KUB radiography and NCCT were reviewed in a blinded manner by a qualified radiologist. Thus, despite the low detection rate of ureteric stones at KUB, this rate is considered the best and is not usually available in emergency settings. Therefore, this paper is recognized as one of the first studies to determine, through a blinded evaluation by a qualified senior radiologist, that KUB should not be utilized as a screening method in emergencies due to the risk of overlooking a considerable number of stones, potentially resulting in complex complications such as fever and AKI.

## Conclusions

KUB radiography should not be used as a triage tool for diagnosing ureteric stones due to the potential adverse outcomes of overlooked obstructed stones. KUB radiography can be reliably used during follow-up, as there is a strong correlation between KUB and NCCT in terms of size, site, and laterality.
